# Efficacy and safety of edaravone dexborneol for acute ischemic stroke: a meta-analysis

**DOI:** 10.3389/fneur.2026.1794547

**Published:** 2026-08-26

**Authors:** Xinxiu Shi, Yukai Wang, Renjie Wang, Xiaohua Shi, Lei Xu

**Affiliations:** 1 Department of Neurology, China-Japan Union Hospital of Jilin University, Changchun, Jilin, China; 2 Departments of Nuclear Medicine, China-Japan Union Hospital of Jilin University, Changchun, Jilin, China

**Keywords:** acute ischemic stroke, edaravone dexborneol, efficacy, meta-analysis, safety

## Abstract

**Objective:**

To evaluate the safety and efficacy of Edaravone Dexborneol in the treatment of acute ischemic stroke (AIS).

**Methods:**

Randomized clinical trials (RCTs) and Prospective cohort study on Edaravone Dexborneol for AIS were retrieved from PubMed, Embase, Cochrane Library, Web of Science, and CNKI. The quality of included studies was assessed using the Cochrane risk of bias tool. Meta-analysis was performed using RevMan 5.2. Efficacy evaluation is based on the changes in the National Institutes of Health Stroke Scale (NIHSS) and mRS scores from baseline to 10–14 days after treatment, as well as the proportion of patients achieving a modified Rankin Scale (mRS) score of 0–1 at 90 days. Safety evaluation is based on the incidence of adverse events during treatment or within 3 months.

**Results:**

The meta-analysis included 54 studies. For efficacy evaluation: 1. Compared with the control group, the edaravone dexborneol (ED) treatment group showed a reduction in NIHSS score of 2.27, which was statistically significant (*I*^2^ = 93%, MD = –2.27, 95% CI:–2.61 to–1.94, *p* < 0.00001). Subgroup analysis based on the mean baseline NIHSS score categorized patients into mild-to-moderate, moderate-to-severe, and severe stroke subgroups. In the mild-to-moderate stroke subgroup: (*I*^2^ = 91%, MD = –1.92, 95% CI:–2.30 to–1.54, *p* < 0.00001.). In the moderate-to-severe stroke subgroup: (*I*^2^ = 94%, MD = −2.76, 95% CI: −3.72 to −1.79, *p* < 0.00001). In the severe stroke subgroup: (*I*^2^ = 80%, MD = −3.50, 95% CI: −4.76 to −2.24, *p* = 0.04). Patients with higher baseline NIHSS scores may have more pronounced functional improvement after ED treatment. 2. At 2 weeks after treatment, the ED group showed a reduction in mRS score of 0.93 compared with the control group, with statistical significance (*I*^2^ = 98%, MD = –0.93, 95% CI:–1.23 to–0.63, *p* < 0.00001). 3. For the proportion of patients achieving mRS = 0–1 at 90 days after treatment, 7 studies were included, with 4,925 patients in the ED group and 3,289 in the control group (*I*^2^ = 57%, RR = 1.10, 95% CI: 1.02 to 1.18, *p* = 0.01). The results indicate a statistically significant functional improvement at 90 days after ED treatment for acute cerebral infarction. Therefore, ED may achieve both short-term and long-term neurological improvement in acute ischemic stroke. For safety evaluation, there was no statistically significant difference in the incidence of adverse events between the ED group and the control group (RR = 0.99, 95% CI: 0.95 to 1.03, *p* = 0.60).

**Conclusion:**

This meta-analysis provides evidence supporting the efficacy and safety of Edaravone Dexborneol for both short- and long-term treatment of acute ischemic stroke. It significantly reduced NIHSS and mRS scores at 10–14 days post-treatment and increased the proportion of patients achieving an mRS score of 0–1 at 90 days. Patients with higher baseline NIHSS scores may derive greater prognostic benefit from edaravone dexborneol therapy. Future multicenter, large-sample clinical studies are still needed to further validate the efficacy and safety of Edaravone Dexborneol.

## 1 Introduction

Stroke affects up to one in five people during their lifetime in some high-income countries and nearly one in two in low-income countries. Globally, it is the second leading cause of death (1). Acute ischemic stroke (AIS) accounts for approximately 60–80% of all strokes. The high incidence and disability rates associated with AIS make it a major public health challenge (2). The core of AIS treatment lies in salvaging the ischemic penumbra, with key therapeutic strategies focusing on restoring blood flow and protecting ischemic brain tissue (3). Although endovascular therapy and intravenous thrombolysis can restore blood flow by recanalizing occluded vessels, their efficacy is limited in some patients due to the narrow therapeutic time window for thrombolysis and the impact of reperfusion injury (4). Cerebroprotective agents represent another crucial therapeutic strategy for AIS. These agents offer multi-target cytoprotection against the ischemic cascade—including excitotoxicity, oxidative stress, inflammation, and apoptosis—thereby mitigating ischemic brain damage, salvaging the penumbra, and preventing reperfusion injury (5, 6). Edaravone, an antioxidant and free radical scavenger approved in Japan in 2001 for AIS treatment, alleviates neuronal and endothelial cell injury by scavenging free radicals, reducing neurotoxicity, and modulating the expression of endothelial and neuronal proteins (7). Dexborneol, a bicyclic monoterpenoid compound, suppresses pro-inflammatory factor expression and protects brain function. Edaravone dexborneol is a dual-target cerebroprotective agent independently developed in China. It is currently administered intravenously or sublingually and exerts neuroprotective effects by reducing oxidative stress from the ischemic cascade, readily crossing the blood–brain barrier, and modulating inflammatory responses, thereby alleviating brain tissue damage and its progression (8–11). The two components of edaravone dexborneol have been shown to be effective in preventing and treating AIS (12, 13). Previous meta-analyses (14, 15) have suggested the efficacy and safety of edaravone dexborneol for AIS. However, these analyses were limited by the number of included studies and were confined to English-language publications. Therefore, this study conducts a meta-analysis incorporating both Chinese and English literature to further investigate the efficacy and safety of edaravone dexborneol in treating AIS, aiming to provide more robust evidence for clinical application and individualized treatment.

## 2 Materials and methods

### 2.1 Study protocol

The aim of this meta-analysis was to observe the clinical efficacy and Safety of edaravone dexborneol in the treatment of AIS. This study has completed registration in PROSPERO (CRD420251271060) 22 Dec 2025 and will be conducted in strict compliance with PRISMA guidelines.

### 2.2 Inclusion and exclusion criteria

Inclusion criteria: (1) Randomized controlled trials (RCTs) or Prospective cohort study; (2) Studies involving patients with acute ischemic stroke (AIS); (3) Experimental group receiving conventional treatment plus edaravone dexborneol (ED), with the control group receiving conventional treatment only or conventional treatment + edaravone; (4) Studies with complete data, where original data are obtainable from the authors; (5) Literature published in English or Chinese; (6) Literature published on or before January 22, 2026; (7) Studies that applied the National Institutes of Health Stroke Scale (NIHSS) to assess efficacy before and after treatment; (8) ED administration regimen of twice daily for a treatment duration of 10–14 days.

Exclusion criteria: (1) Studies with obvious logical errors; (2) Studies with non-rigorous inclusion/exclusion criteria that could lead to biased results; (3) Studies lacking objective reference standards; (4) Studies with a follow-up success rate of less than 80%; (5) Studies where authors have potential conflicts of interest; (6) Studies lacking statements regarding ethical approval or informed consent; (7) Studies where ED treatment duration was less than 10 days or administered once daily; (8) Publication types including abstracts, conference proceedings, reviews, or animal studies.

### 2.3 Search strategy

Databases such as PubMed, Web of Science, Cochrane, Embase and CNKI were searched for clinical studies on edaravone dexborneol in the treatment of AIS from the date of establishment to January-20-2026 using medical subject heading combined entry terms. Keywords included edaravone dexborneol, acute ischemic stroke.

Take Pubmed’s query box as an example (((((((acute ischemic stroke[Title/Abstract]) OR (Ischemic Stroke[Title/Abstract])) OR (Brain Ischemia[Title/Abstract])) OR (Cerebral Infarction[Title/Abstract])) OR (acute cerebral infarction[Title/Abstract])) OR (acute brain ischemia[Title/Abstract])) OR (acute cerebrovascular accident[Title/Abstract])) OR (acute ischemic stroke[MeSH Major Topic]) AND (edaravone dexborneol[Title/Abstract]).

### 2.4 Literature screening and data extraction

Two researchers independently screened the literature and extracted data. For studies with discrepancies, decisions were strictly based on the inclusion and exclusion criteria, and expert consultation was sought to confirm the screening outcome. Relevant data were extracted from the included studies using a custom form, including: (1) basic article information (e.g., author, publication year); (2) participant and methodology details (e.g., sample size, age); (3) intervention specifics (edaravone dexborneol treatment regimen and duration); (4) outcome measures: 1. Efficacy outcome measures included changes in NIHSS and mRS scores from baseline to 10–14 days after treatment, as well as the proportion of patients achieving an mRS score of 0–1 at 90 days. Safety evaluation was based on the incidence of adverse events during treatment or within 3 months.

### 2.5 Literature quality evaluation

Study quality was assessed using the Cochrane Risk of Bias tool (RoB 2.0) (16), which evaluates six domains: random sequence generation, allocation concealment, blinding of participants and personnel, blinding of outcome assessment, incomplete outcome data, and selective reporting. Each domain was judged as “low risk,” “high risk,” or “unclear risk.” Two reviewers independently performed the assessments, and any disagreements were resolved through consensus.

### 2.6 Statistical analysis

Meta-analysis and graph generation were performed using RevMan software (version 5.2). The relative risk (RR) was chosen as the effect measure for dichotomous data, and the weighted mean difference (WMD) for continuous data. An I^2^ statistic ≤ 50% indicated acceptable heterogeneity, and a fixed-effects model was applied. An *I*^2^ statistic > 50% indicated significant heterogeneity, and a random-effects model was used. A *p*-value < 0.05 was considered statistically significant.

A subgroup analysis based on baseline NIHSS scores was conducted to explore the efficacy difference of edaravone dexborneol versus control across different stroke severities. For outcome measures with ≥10 included studies, publication bias was assessed using funnel plots (where basic bilateral symmetry indicates a low risk of bias) and Begg’s test (*α* = 0.05). Sensitivity analysis was performed using the leave-one-out method in Stata 18 to evaluate the influence of individual study results on the overall pooled estimate.

## 3 Results

### 3.1 Literature screening results

**Figure 1:**
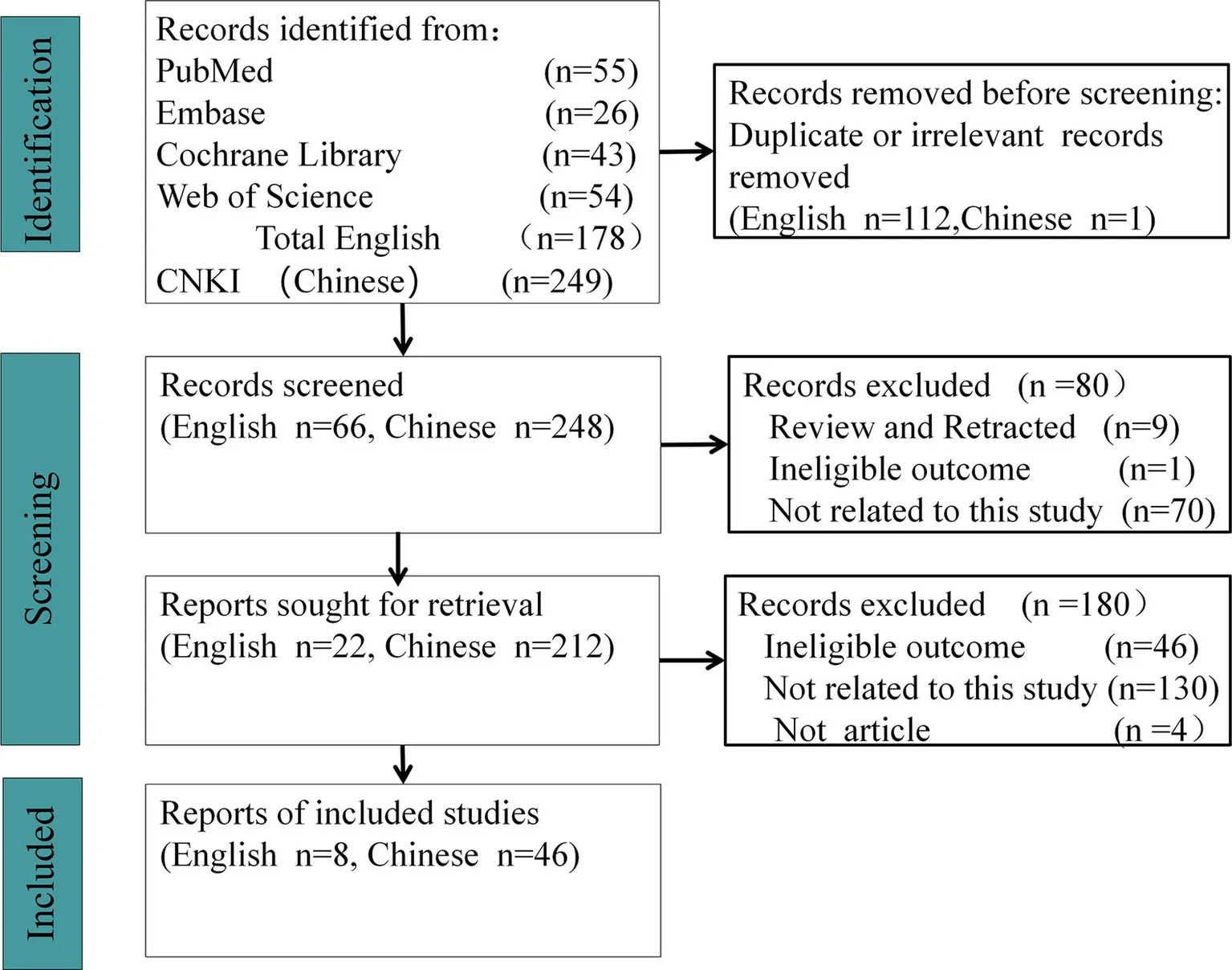
Screening process of literature.

**Figure 2:**
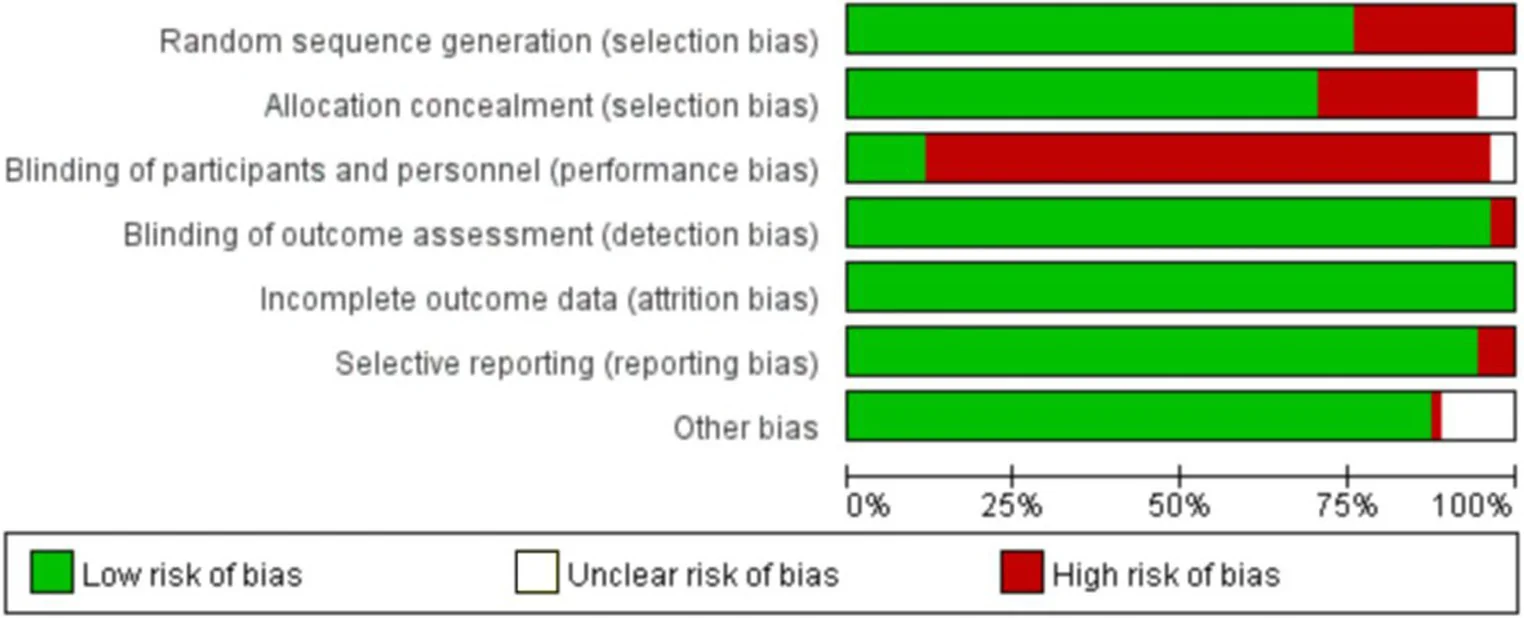
Risk assessment results from the literature.

A total of 427 records related to edaravone dexborneol treatment were initially identified through searches of PubMed, Web of Science, Embase, Cochrane Library, and CNKI. After deduplication, manual review, and application of the screening criteria, 54 studies (8, 11, 17–68) were ultimately included in the meta-analysis (see the literature selection flowchart in Figure 1). The quality assessment of the included studies indicated a predominantly low risk of bias, suggesting their high reference value (Figure 2).

### 3.2 Basic characteristics of subjects

The 54 articles enrolled involved a total of 12,631 subjects, among which 7,142 patients were treated with ED (the experimental group), and 5,489 patients were treated with conventional treatment (the control group) (Table 1).

**Table 1:** The basic information of the included studies.

Study	EP (N)	CP (N)	EP(Age^a^)	CP(Age^a^)	EP (TM)	CP (TM)	DT	EP(NIHSS^a^ at baseline)	CP(NIHSS^a^ at baseline)	EP(NIHSS^a^ after treatment)	CP(NIHSS^a^ after treatment)	EP(Changes of NIHSS^b^)	CP(Changes of NIHSS^b^)	EP (AE^c^)	CP (AE^c^)
2019 Xu J (8)	74	73	59.11 ± 8.95	59.71 ± 8.33	①	④	14	6.99 ± 3.31	7.01 ± 3.41	/	/	−2.58 ± 3.42	−3.03 ± 3.64	/	/
2021 Xu J(17)	585	580	62.96 (55.38–68.96)	62.86(55.72–70.12)	①	④	14	6.00(5–9)	6.00(5–8)	/	/	−2.94 ± 2.53	−2.54 ± 3.01	558	559
2021 Zhang L (18)	40	40	62.5 ± 3.8	59.5 ± 3.7	①	④	14	11.4 ± 2.6	12.5 ± 2.5	3.1 ± 3.2	4.9 ± 2.5	−8.3 ± 2.95	−7.6 ± 2.50	1	2
2021 Zhang Y (19)	30	30	63.45 ± 3.89	64.32 ± 4.23	①	④	14	18.61 ± 2.21	18.31 ± 2.11	4.67 ± 0.98	9.87 ± 1.01	−13.94 ± 1.92	−8.44 ± 1.83	/	/
2022 Tong C (20)	41	41	57.80 ± 4.35	57.65 ± 4.29	①	④	14	19.10 ± 2.52	19.01 ± 2.45	6.05 ± 1.31	9.63 ± 1.72	−13.05 ± 2.18	−9.38 ± 2.18	1	4
2022 Wang H (21)	44	44	65.36 ± 4.28	65.71 ± 4.56	①	④	14	8.37 ± 2.69	8.58 ± 2.76	4.61 ± 1.52	6.03 ± 1.98	−3.76 ± 2.34	−2.55 ± 2.46	3	6
2022 Lu P (22)	45	45	62.98 ± 7.05	63.42 ± 7.76	①	④	14	18.80 ± 2.48	18.84 ± 2.58	9.53 ± 1.25	11.20 ± 1.62	−9.27 ± 2.15	−7.64 ± 2.26	2	3
2022 Xia X (23)	46	46	/	/	①	④	14	9.32 ± 2.12	9.45 ± 3.19	4.23 ± 0.69	5.96 ± 0.85	−5.09 ± 1.87	−3.49 ± 2.86	5	9
2023 Wu D (24)	45	45	59.64 ± 9.43	58.91 ± 10.14	①	④	14	18.07 ± 4.26	17.78 ± 4.30	11.40 ± 3.54	13.18 ± 3.88	−6.67 ± 3.95	−4.60 ± 4.11	4	9
2023 Deng H (25)	38	38	65.37 ± 5.84	65.48 ± 5.91	①	④	14	18.82 ± 2.48	18.78 ± 2.51	9.51 ± 1.24	11.24 ± 1.63	−9.31 ± 2.15	−7.54 ± 2.21	3	10
2023 Li J (26)	60	60	61.62 ± 10.54	61.58 ± 10.49	①	④	14	18.74 ± 2.19	18.79 ± 2.17	9.46 ± 1.07	11.36 ± 1.84	−9.28 ± 1.90	−7.43 ± 2.03	3	4
2023 Jiang J (27)	38	38	52.62 ± 2.64	52.45 ± 2.56	①	④	14	13.33 ± 1.21	13.26 ± 1.12	2.86 ± 0.46	4.44 ± 0.78	−10.47 ± 1.06	−8.82 ± 0.99	/	/
2023 Hu X (28)	69	73	65.8 ± 11.0	66.6 ± 12.7	①	⑤	10–14	10.0 ± 6.1	10.6 ± 6.4	/	/	−5.13 ± 5.29	−3.26 ± 4.00	14	29
2024 Li D (29)	67	56	60.33 ± 9.57	60.73 ± 7.9	①	⑤	14	/	/	/	/	−9.76 ± 2.75	−6.26 ± 3.83	7	5
2024 Shao S (30)	34	34	60.85 ± 3.42	61.14 ± 3.68	①	④	14	17.75 ± 2.11	17.63 ± 2.15	5.22 ± 0.96	9.35 ± 1.27	−12.53 ± 1.83	−8.28 ± 1.87	4	5
2024 Chen W (31)	41	44	65.15 ± 10.82	68.06 ± 11.55	①	⑤	14	5 (4–9)	5 (4–10)	2 (1–4)	4 (2–8)	−3 ± 3.23	−1 ± 4.44	/	/
2024 Fu Y (11)	450	464	64.1 (56.0–69.8)	64.6 (57.1–71.4)	②	⑥	14	7 (7–8)	7 (7–8)			−3 ± 5.41	−3 ± 5.49	405	418
2025 Wang B (32)	46	46	57.13 ± 4.61	59.96 ± 4.55	①	⑤	14	12.37 ± 3.36	12.31 ± 3.45	5.83 ± 1.72	7.24 ± 2.16	−6.54 ± 2.91	−5.07 ± 3.02	/	/
2025 Gai B (33)	50	50	72.13 ± 5.69	71.86 ± 5.31	①	⑤	14	20.16 ± 2.08	20.19 ± 2.06	7.27 ± 0.83	13.73 ± 1.65	−12.89 ± 1.81	−6.46 ± 1.89		
2025 Liu B (34)	74	74	57.75 ± 8.14	58.32 ± 8.61	①	⑤	14	15.37 ± 2.14	15.51 ± 2.23	7.27 ± 1.28	11.06 ± 1.81	−8.10 ± 1.87	−4.45 ± 2.05	6	7
2025 Liu C (35)	35	34	60.25 ± 8.12	62.13 ± 7.95	①	⑤	14	15. 26 ± 3. 15	15.32 ± 3.08	7.12 ± 2.05	9.68 ± 2.51	−8.14 ± 2.77	−5.64 ± 2.84	/	/
2025 Ye C (36)	50	50	57.46 ± 5.87	58.01 ± 5.88	①	⑤	14	12.16 ± 3.31	11.98 ± 3.22	2.04 ± 0.56	5.87 ± 1.64	−10.12 ± 3.07	−6.11 ± 2.79	3	10
2025 Li C (37)	54	52	63.37 ± 7.51	63.54 ± 7.68	①	⑤	14	15.72 ± 1.34	15.58 ± 1.39	5.31 ± 0.78	6.28 ± 0.84	−10.41 ± 1.17	−9.30 ± 1.21	6	4
2025 Gong D (38)	61	61	56.32 ± 3.24	57.14 ± 3.18	①	④	14	14.26 ± 2.75	13.87 ± 2.49	7.83 ± 1.29	8.79 ± 1.41	−6.43 ± 2.38	−5.08 ± 2.16	4	6
2025 Liu D (39)	40	40	61.73 ± 3.51	62.05 ± 3.75	①	⑤	14	15.74 ± 1.23	16.12 ± 1.41	5.24 ± 0.57	8.46 ± 0.84	−10.50 ± 1.07	−7.66 ± 1.23	/	/
2025 Ma G (40)	3,017	1,384	65(56–72)	66(58–74)	①	⑤	14	3 (2–7)	3(1–5)	/	/	−2 ± 4.2	−1 ± 4.75	**12**	8
2025 Sun G (41)	48	48	71.50 ± 10.50	70.05 ± 9.95	①	⑤	10	12.93 ± 4.84	12.99 ± 3.18	5.23 ± 1.25	8.92 ± 1.38	−7.70 ± 4.35	−4.07 ± 2.76	/	/
2025 Yang H (42)	40	40	59.51 ± 3.54	59.56 ± 3.58	①	⑤	15	19. 92 ± 2. 03	19. 87 ± 1. 98	8.44 ± 0.67	12.55 ± 1.55	−11.48 ± 1.79	−7.32 ± 1.80	/	/
2025 Dong H (43)	71	71	58.37 ± 7.39	57.56 ± 7.29	①	⑤	14	12.61 ± 2.33	12.17 ± 2.19	5.75 ± 1.28	9.94 ± 1.27	−6.86 ± 2.02	−2.23 ± 1.90	13	11
2025 Wang H (44)	40	40	60.50 ± 4.63	60.60 ± 4.70	①	⑤	14	12.72 ± 2.40	12.60 ± 2.3	5.78 ± 1.43	7.53 ± 1.90	−6.94 ± 2.09	−5.07 ± 2.13	9	7
2025 Hu H (45)	30	30	60.28 ± 1.27	60.56 ± 1.55	①	⑤	14	18.96 ± 2.38	19.62 ± 2.39	10.46 ± 3.36	13.56 ± 3.58	−8.50 ± 2.99	−6.06 ± 3.16	1	8
2025 Ding J (46)	48	48	65.93 ± 6.14	67.02 ± 6.87	①	⑤	14	18.33 ± 2.45	19.04 ± 2.26	7.68 ± 1.95	9.12 ± 2.07	−10.65 ± 2.24	−9.92 ± 2.17	7	10
2025 Lu J (47)	46	46	62.14 ± 10.35	61.79 ± 10.27	①	⑤	14	14.97 ± 2.31	15.12 ± 2.39	7.10 ± 1.35	10.86 ± 1.74	−7.87 ± 2.01	−4.26 ± 2.14	/	/
2025 Gao J (48)	40	40	60.69 ± 9.14	61.47 ± 9.28	①	⑤	14	38.15 ± 14.42	38.12 ± 14.36	19.42 ± 4.17	23.56 ± 9.28	−18.73 ± 12.85	−14.56 ± 12.61	2	8
2025 Gu J (49)	59	58	63.96 ± 2.34	64.12 ± 2.32	①	⑤	14	14.83 ± 2.51	15.49 ± 2.45	7.61 ± 1.05	8.27 ± 1.18	−7.22 ± 2.18	−7.22 ± 2.12	8	6
2025 Shi J (50)	30	30	47.69 ± 7.24	46.98 ± 7.02	①	⑤	14	31.21 ± 5.31	30.19 ± 5.76	9.41 ± 1.82	12.81 ± 2.18	−21.80 ± 4.67	−17.38 ± 5.04	4	3
2025 Chen L (51)	30	30	51.23 ± 9.76	50.75 ± 9.84	①	⑤	14	32.76 ± 5.23	32.74 ± 5.24	17.02 ± 2.21	24.03 ± 3.22	−15.74 ± 4.55	−8.71 ± 4.58	5	3
2025 Zhang L (52)	52	52	63.15 ± 6.64	61.84 ± 7.38	①	⑤	14	15.61 ± 5.36	15.58 ± 5.21	8.32 ± 2.67	12.56 ± 4.82	−7.29 ± 4.64	−3.02 ± 5.03	5	4
2025 Hu L (53)	40	40	67.68 ± 7.31	67.52 ± 7.26	①	⑤	14	12.63 ± 2.71	12.52 ± 2.69	6.72 ± 1.05	8.93 ± 1.14	−5.91 ± 2.37	−3.59 ± 2.34	4	2
2025 Wu L (54)	30	30	60.21 ± 16.89	63.06 ± 14.61	①	⑤	14	5.39 ± 0.43	5.43 ± 0.49	3.11 ± 0.25	4.38 ± 0.37	−2.28 ± 0.37	−1.05 ± 0.44	/	/
2025 Li L (55)	35	35	63.26 ± 1.48	63.39 ± 1.57	①	⑤	14	17.53 ± 2.16	17.61 ± 2.34	7.86 ± 1.49	9.16 ± 1.66	−9.67 ± 1.92	−8.45 ± 2.09	4	6
2025 Zhao P (56)	59	58	63.78 ± 9.46	64.14 ± 8.95	①	⑤	14	8.92 ± 1.17	8.89 ± 1.25	2.87 ± 0.52	4.12 ± 0.63	−6.05 ± 1.02	−4.77 ± 1.08	5	4
2025 Wang R (57)	49	49	65.03 ± 2.02	65.04 ± 2.05	①	⑤	14	32.15 ± 0.11	32.13 ± 0.10	16.12 ± 0.23	18.33 ± 0.42	−16.03 ± 0.20	−13.80 ± 0.38	4	3
2025 Yao R (58)	106	106	61.91 ± 5.94	62.05 ± 5.89	①	⑤	14	14. 78 ± 2. 33	15. 03 ± 2. 21	8.67 ± 1.23	10.82 ± 1.	−6.11 ± 2.02	−4.21 ± 1.97	6	8
2025 Hu S (59)	55	55	72.65 ± 9.95	71.98 ± 9.89	①	⑤	14	8.25 ± 6.07	8.51 ± 5.93	0.00(0.00–2)	2.00(0.00–10)	−8.25 ± 5.48	−6.51 ± 6.79	4	2
2025 Xia X (60)	37	36	63.96 ± 2.34	64.12 ± 2.32	①	⑤	14	14.53 ± 2.51	14.49 ± 2.45	6.61 ± 1.05	10.27 ± 1.18	−7.92 ± 2.18	−4.22 ± 2.12	4	3
2025 Xu X (61)	45	45	59.12 ± 7.14	58.85 ± 6.73	③	⑤	14	17.38 ± 2.24分	17.13 ± 2.09	8.84 ± 1.42	11.15 ± 1.67	−9.28 ± 1.90	−7.43 ± 2.03	3	3
2025 Xu XR (62)	36	36	61.14 ± 3.55	60.38 ± 3.47	①	⑤	14	17.52 ± 2.85	18.36 ± 3.52	12.22 ± 1.35	13.83 ± 1.86	−5.30 ± 2.47	−4.53 ± 3.05	2	4
2025 Guo Y (63)	49	49	64.38 ± 4.52	64.38 ± 4.52	①	⑤	10	15.93 ± 2.76	16.09 ± 2.79	9.31 ± 2.27	11.62 ± 2.38	−6.62 ± 2.55	−4.47 ± 2.61	5	3
2025 Hong Y (64)	50	50	66.08 ± 6.91	65.02 ± 8.45	①	⑤	14	8(6–9)	7(6–9)	5(3–6)	6(4–7)	−3 ± 2.22	−1 ± 2.22	2	1
2025 Wang Y (65)	62	62	66.21 ± 5.36	65.87 ± 5.11	①	⑤	14	11. 24 ± 3. 17	11.15 ± 3.04	5.14 ± 1.28	7.23 ± 1.41	−6.10 ± 2.76	−3.92 ± 2.63	12	9
2025 Zhou Y (66)	41	41	87.25 ± 3.01	87.42 ± 3.06	①	⑤	14	20.46 ± 2.25	21.18 ± 2.13	11.18 ± 1.28	14.42 ± 1.53	−9.28 ± 1.95	−6.76 ± 1.90	5	3
2025 Yue (67)	50	50	66.39 ± 6.97	66.26 ± 6.84	①	⑤	14	17.69 ± 4.62	17.58 ± 3.83	8.56 ± 1.17	11.14 ± 1.45	−9.13 ± 4.16	−6.44 ± 3.35	/	/
2026 Wang C (68)	690	672	67.0(57.0–73.0)	67.0(58.0–73.0)	①	⑤	10–14	15.0(12–19)	15.0(11–19)	/	/	−7.1 (7.7)	−7.0 (8.3)	188	173

N, number; NIHSS, National Institutes of Health Stroke Scale; AE, adverse event; EP, Experimental group; CP, Control group; TM,treatment methods; DT, Duration of treatment/d; a, (mean ± SD)，median (IQR); b, (mean ± SD); c, during treatment or within 90 days; ① 37.5 mg (30 mg of edaravone and 7.5 mg of (+)-dexborneol) injection. Twice a day+conventional treatment; ② sublingual edaravone dexborneol (edaravone, 30 mg; dexborneol, 6 mg), twice a day + conventional treatment; ③ 37.5 mg (30 mg of edaravone and 7.5 mg of (+)-dexborneol) injection. Once a day + conventional treatment; ④ 30 mg/dose (edaravone, 30 mg) injection, twice a day + conventional treatment; ⑤ conventional treatment; ⑥ sublingual placebo (edaravone, 0 mg; dexborneol,60 μg) twice a day + conventional treatment. Presentation of baseline characteristics of the included studies.

### 3.3 Clinical efficacy analysis of edaravone dexborneol in the treatment of AIS

#### 3.3.1 Evaluation of the therapeutic effect of edaravone dexborneol-changes in NIHSS before and after treatment

**Figure 3:**
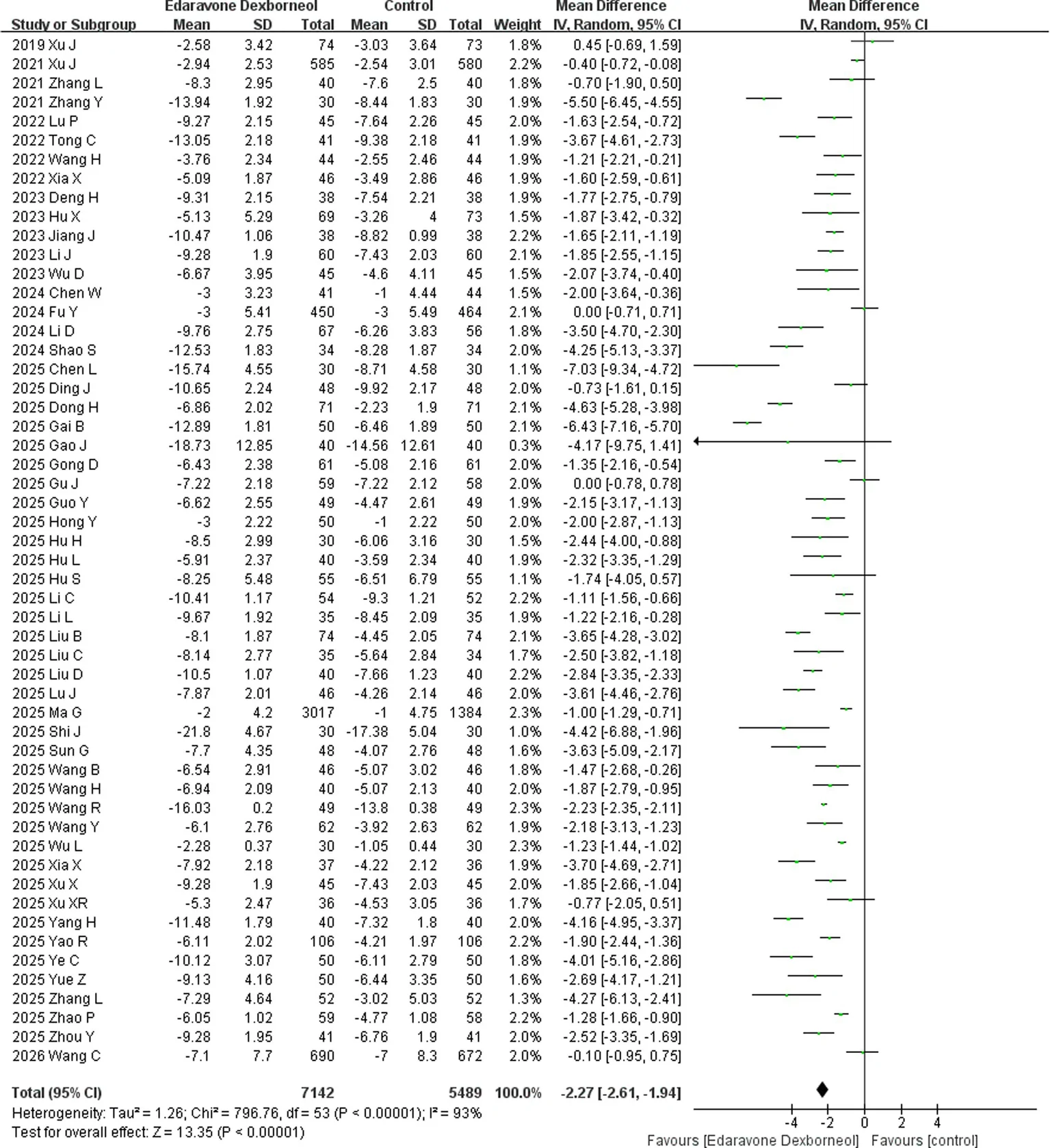
Change in NIHSS score before and after treatment in the ED group versus the control group.

Fifty-four studies reported NIHSS scores. Significant heterogeneity was observed among the studies (*I*^2^ = 93%, *p* < 0.00001); therefore, a random-effects model was used for analysis. The results showed that the NIHSS score reduction was significantly greater in the experimental group than in the control group, with a mean difference (MD) of −2.27 points (95% CI = −2.61 to −1.94, *p* < 0.00001) (Figure 3).

#### 3.3.2 Subgroup analysis of changes in NIHSS before and after treatment

A meta-analysis of changes in NIHSS scores before and after treatment was conducted across 54 studies, revealing significant heterogeneity (*I*^2^ = 93%, *p* < 0.00001; Figure 3). Due to the high heterogeneity, a subgroup analysis was performed (grouping details are presented in Table 2).

**Table 2:** The basic information of subgroup analyses.

Study	EP (TM)	CP (TM)	DT	1–4 indicate minor (1); 5-15, moderate (2); 16-20 moderate-to-severe (3); and 21-42 severe stroke (4)	CP Treatment: conventional treatment (1); edaravone + conventional treatment (2)	mRS2W (1); mRS3m (2)	EP(mRS)^d,e^	EP (N)	CP (mRS)^d,e^	CP (N)	Study quality (Random enrollment Yes = 1, No = 0)	Reperfusion therapy (Yes = 1, No = 0)	Onset time < 24 h (Yes = 1; No = 0)	Single group sample size ≥ 50 (Yes = 1 No = 0)
2019 Xu J (8)	①	④	14	2	2	2	45/74^d^	74	51/73	73	1	0	0	1
2021 Xu J (17)	①	④	14	2	2	2	393/585	585	342/580	580	1	0	0	1
2021 Zhang L (18)	①	④	14	2	2	1	−5.0 ± 0.5 ^e^	40	−1.5 ± 0.5	40	1	1	1	0
2021 Zhang Y (19)	①	④	14	3	2	1	−1.95 ± 0.68	30	−1.00 ± 0.72	30	1	1	1	0
2022 Lu P (22)	①	④	14	3	2	1	−1.74 ± 0.68	45	−1.31 ± 0.63	45	1	1	1	0
2022 Tong C (20)	①	④	14	3	2	1	−2.52 ± 0.59	41	−1.56 ± 0.53	41	1	1	1	0
2022 Wang H (21)	①	④	14	2	2	1	−1.27 ± 1.10	44	−0.16 ± 1.17	44	1	0	0	0
2022 Xia X (23)	①	④	14	2	2	1	−1.20 ± 0.56	46	−0.73 ± 0.48	46	1	0	0	0
2023 Deng H (25)	①	④	14	3	2		/	38	/	38	1	1	1	0
2023 Hu X (28)	①	⑤	10–14	2	1	2	90 Days,44/69	69	37/73	73	1	0	0	1
2023 Jiang JR (27)	①	④	14	2	2	1	−5.16 ± 0.49	38	−1.50 ± 0.53	38	1	0	0	0
2023 Li J (26)	①	④	14	3	2		/	60	/	60	1	1	1	1
2023 Wu D (24)	①	④	14	3	2	1	−1.18 ± 0.64	45	−0.69 ± 0.55	45	1	0	0	0
2024 Chen W (31)	①	⑤	14	2	1	2	29/41	41	21/44.	44	0	0	0	0
2024 Fu Y (11)	②	⑥	14	2	1	2	290/450	450	254/464	464	1	0	0	1
2024 Li D (29)	①	⑤	14		1		/	67	/	56	0	1	1	1
2024 Shao S (30)	①	④	14	3	2	1	−1.27 ± 0.49	34	−0.54 ± 0.57	34	1	0	1	0
2025 Chen L (51)	①	⑤	14	4	1	1	−1.75 ± 0.90	30	−1.34 ± 0.92	30	1	1	1	0
2025 Ding J (46)	①	⑤	**14**	**3**	**1**		**/**	**48**	**/**	**48**	**1**	**1**	**1**	0
2025 Dong H (43)	①	⑤	14	2	1		/	71	/	71	1	1	1	1
2025 Gai B (33)	①	⑤	14	3	**1**		/	50	/	50	1	0	/	1
2025 Gao J (48)	①	⑤	14	4	1		/	40	/	40	1	0	1	0
2025 Gong D (38)	①	④	14	2	2		/	61	/	61	1	0	/	1
2025 Gu J (49)	①	⑤	14	2	1	1	−1.80 ± 0.32	59	−1.05 ± 0.32	58	1	0	1	1
2025 Guo Y (63)	①	⑤	10	2	1	1	−1.56 ± 1.33	49	−0.86 ± 1.28	49	0	1	1	0
2025 Hong Y (64)	①	⑤	14	2	1		/	50	/	50	0	0	0	1
2025 Hu H (45)	①	⑤	14	3	1		/	30	/	30	0	1	1	0
2025 Hu L (53)	①	⑤	14	2	1		/	40	/	40	0	0	1	0
2025 Hu S (59)	①	⑤	14	2	1	1	−3.35 ± 1.65	55	−0.98 ± 3.35	55	0	1	1	1
2025 Li C (37)	①	⑤	14	2	1	1	−1.64 ± 0.37	54	−1.05 ± 0.38	52	0	1	1	1
2025 Li L (55)	①	⑤	14	3	1		/	35	/	35	0	1	1	0
2025 Liu B (34)	①	⑤	14	2	1		/	74	/	74	1	0	0	1
2025 Liu C (35)	①	⑤	14	2	1		/	35	/	34	1	0	0	0
2025 Liu D (39)	①	⑤	14	2	1		/	40	/	40	1	1	1	0
2025 Lu J (47)	①	⑤	14	2	1	1	−1.8 ± 0.77	46	−0.86 ± 0.86	46	1	0	1	0
2025 Ma G (40)	①	⑤	14	1	1	2	2071/3017;	3,017	914/1384	1,384	1	0	1	1
2025 Shi J (50)	①	⑤	14	4	1		−2.59 ± 0.84	30	−2.17 ± 0.92	30	1	0	0	0
2025 Sun G (41)	①	⑤	10	2	1		/	48	/	48	0	1	1	0
2025 Wang B (32)	①	⑤	14	2	1	1	−1.16 ± 0.45	46	−0.89 ± 0.47	46	1	0	/	0
2025 Wang H (21)	①	⑤	14	2	1	1	−2.33 ± 0.55	40	−1.59 ± 0.66	40	0	0	0	0
2025 Wang R (57)	①	⑤	14	4	1	1	−1.70 ± 0.23	49	−0.86 ± 0.28	49	0	0	1	0
2025 Wang Y (65)	①	⑤	14	2	1	1	−2.03 ± 0.86	62	−1.59 ± 0.79	62	1	1	1	1
2025 Wu L (54)	①	⑤	14	2	1	1	−0.90 ± 0.25	30	−0.46 ± 0.23	30	1	1	1	0
2025 Xia X (60)	①	⑤	14	2	1	1	−1.77 ± 0.30	37	−1.08 ± 0.34	36	1	0	1	0
2025 Xu XL (61)	③	⑤	14	3	1		/	45	/	45	1	0	1	0
2025 Xu XR (62)	①	⑤	14	3	1		/	36	/	36	1	0	1	0
2025 Yang H (42)	①	⑤	15	3	1		/	40	/	40	1	1	1	0
2025 Yao R (58)	①	⑤	14	2	1	1	−1.10 ± 0.61	106	−0.54 ± 0.65	106	1	0	1	1
2025 Ye C (36)	①	⑤	14	2	1		/	50	/	50	1	0	1	1
2025 Yue Z (67)	①	⑤	14	3	1	1	−2.69 ± 0.86	50	−2.13 ± 0.79	50	1	1	1	1
2025 Zhang L (52)	①	⑤	14	2	1	1	−1.90 ± 0.64	52	−1.47 ± 0.69	52	1	0	1	1
2025 Zhao P (56)	①	⑤	14	2	1		/	59	/	58	1	0	0	1
2025 Zhou Y (66)	①	⑤	14	4	1	1	−1.87 ± 0.65	41	−0.76 ± 0.77	41	1	0	0	0
2026 Wang C (68)	①	⑤	10–14	2	1	2	276/689	690	255/671	672	1	1	1	1

^d^(n/N), ^e^(mean ± S). Presentation of baseline characteristics of the clinical studies included in the subgroup analysis.

**Figure 4:**
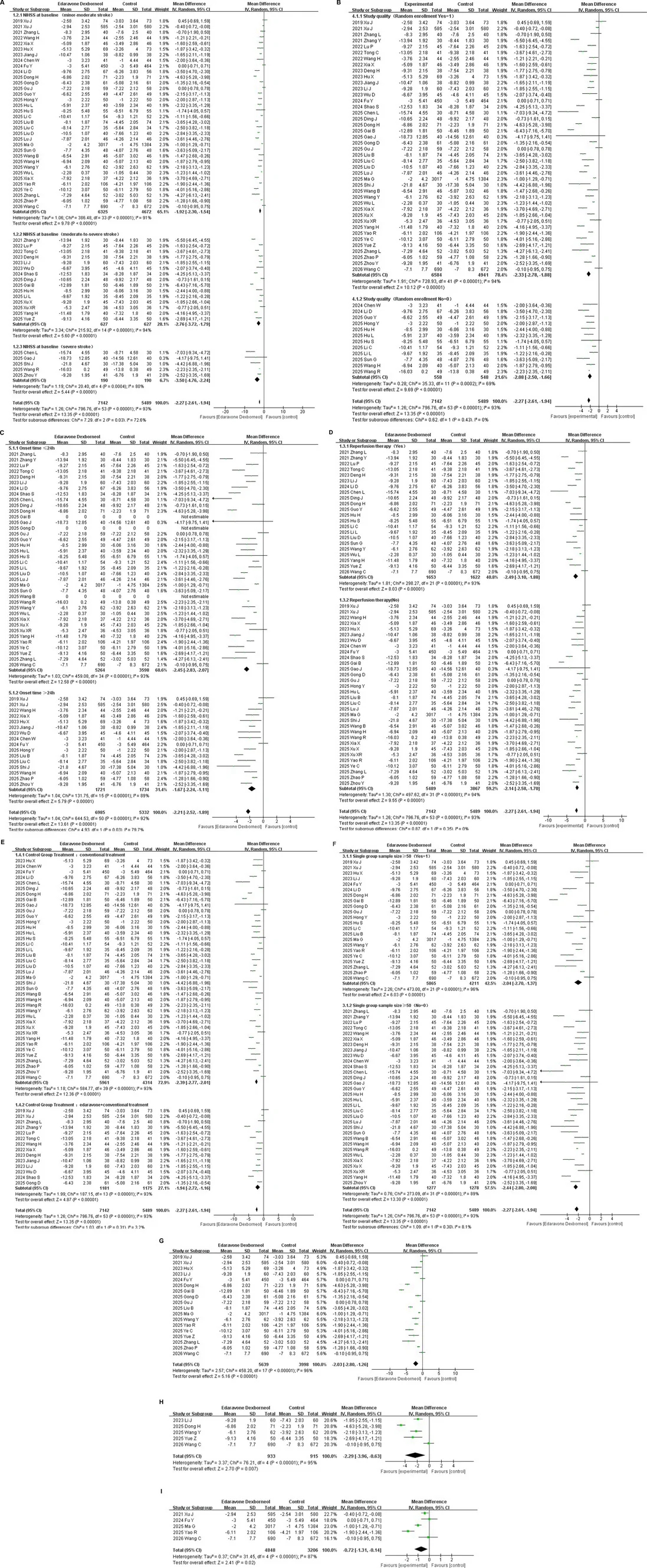
**(A)** Subgroup analysis stratified by baseline NIHSS score differences. **(B)** Subgroup analysis based on whether enrollment was randomized. **(C)** Subgroup analysis based on whether the time from symptom onset was within 24 h. **(D)** Subgroup analysis based on whether reperfusion therapy was administered. **(E)** Subgroup analysis based on the treatment modality in the control group. **(F)** Subgroup analysis based on whether the sample size per group was ≥0. **(G)** Forest plot of pre- and post-treatment NIHSS changes in studies with a sample size of ≥50 per group and randomized enrollment. **(H)** Forest plot of pre- and post-treatment NIHSS changes in studies meeting three criteria: sample size >50 per group, randomized enrollment, and reperfusion therapy. **(I)** Forest plot of pre- and post-treatment NIHSS changes in studies with a sample size of ≥100 per group.

The NIHSS has a total score of 42, with higher scores indicating more severe neurological deficits. According to established classifications (69), scores of 1–4 indicate minor stroke; 5–15, moderate stroke; 16–20, moderate-to-severe stroke; and 21–42, severe stroke. Based on the mean baseline NIHSS score, patients were categorized into mild-to-moderate, moderate-to-severe, and severe stroke subgroups for analysis (Figure 4A). In the mild-to-moderate stroke subgroup, 34 studies were included, with 6,325 patients in the edaravone dexborneol group and 4,672 in the control group [*I*^2^ = 91%, MD = −1.92, 95% CI = −2.30 to −1.54, *p* < 0.00001] (Figure 4A). In the moderate-to-severe stroke subgroup, 15 studies were included, with 627 patients in the edaravone dexborneol group and 627 in the control group [*I*^2^ = 94%, MD = −2.76, 95% CI = −3.72 to −1.79, *p* < 0.00001] (Figure 4A). In the severe stroke subgroup, 3 studies were included, with 190 patients in the edaravone dexborneol group and 190 in the control group [*I*^2^ = 80%, MD = −3.50, 95% CI = −4.76 to −2.24, *p* = 0.04] (Figure 4A). These findings suggest that patients with higher baseline NIHSS scores may experience more pronounced improvement in neurological deficits after ED treatment (the changes in NIHSS before and after treatment in the mild-to-moderate, moderate-to-severe, and severe stroke subgroups were −1.92, –2.76, and −3.50). The *I*^2^ value was notably lower in the severe stroke subgroup, and the difference among the three subgroups was significant (*p* = 0.03), indicating that heterogeneity may be related to differences in baseline NIHSS scores across the included populations. Nevertheless, regardless of stroke severity at enrollment, the therapeutic effect of edaravone dexborneol was statistically significant in all subgroups.

A subgroup analysis based on whether enrollment was randomized was performed (Figure 4B). Randomized enrollment was considered a low risk of bias, while non-randomized enrollment was considered a high risk of bias. Forty-two studies with randomized enrollment, including 6,584 patients in the edaravone dexborneol group and 4,941 in the control group, showed a pooled MD of −2.33 (*I*^2^ = 94, 95% CI:–2.78 to–1.88, *p* < 0.00001). Twelve studies with non-randomized enrollment, including 558 patients in the edaravone dexborneol group and 548 in the control group, showed a pooled MD of −2.08 (*I*^2^ = 93, 95% CI:–2.50 to–1.66, *p* < 0.00001). The difference between the two subgroups was not significant (*p* = 0.31), suggesting that the enrollment method was not strongly associated with heterogeneity. Nonetheless, regardless of whether enrollment was randomized, the treatment effect of edaravone dexborneol remained statistically significant.

A subgroup analysis based on whether the time from symptom onset was within 24 h was performed (Figure 4C). Forty-eight studies with onset within 24 h (5,264 patients in the edaravone dexborneol group vs. 3,598 in the control group) showed [*I*^2^ = 93%, MD = –2.45, 95% CI:–2.83 to–2.07, *p* < 0.00001]; 16 studies with onset beyond 24 h (1,721 patients in the edaravone dexborneol group vs. 1,734 in the control group) showed [I^2^ = 89%, MD = −1.67, 95% CI:–2.24 to–1.11, *p* < 0.00001]. The heterogeneity index varied, and the difference between the two subgroups was significant (*p* = 0.03), suggesting that onset time may be a source of heterogeneity. However, regardless of whether onset was within or beyond 24 h, the edaravone dexborneol group demonstrated significant benefit, with statistical significance.

A subgroup analysis based on the treatment modality in the control group was performed (Figure 4E). In 14 studies where the control group received conventional treatment plus edaravone, the edaravone dexborneol group included 1,181 patients and the control group included 1,175 patients [*I*^2^ = 93%, MD = –1.94, 95% CI = –2.72 to–1.16, *p* < 0.00001]. In 40 studies where the control group received conventional treatment alone, the edaravone dexborneol group included 5,961 patients and the control group included 4,314 patients [*I*^2^ = 93%, MD = −2.39, 95% CI = −2.77 to −2.01, *p* < 0.00001]. The difference between the two subgroups was not significant (*p* = 0.31). The treatment modality was not a source of heterogeneity. Nonetheless, regardless of the treatment received by the control group, the edaravone dexborneol group demonstrated significant benefit, with statistical significance.

A subgroup analysis based on whether the sample size per group was ≥50 patients was performed (Figure 4F). Twenty-two studies with ≥50 patients per group (5,865 in the edaravone dexborneol group vs. 4,211 in the control group) showed [I^2^ = 96%, MD = −2.04, 95% CI: −2.70 to −1.37, *p* < 0.00001]; 30 studies with <50 patients per group (1,277 in the edaravone dexborneol group vs. 1,278 in the control group) showed [I^2^ = 89%, MD = −2.44, 95% CI: −2.80 to −2.08, *p* < 0.00001]. Although the heterogeneity index differed between the two subgroups, the difference between subgroups was not significant (*p* = 0.30). A sample size of ≥50 per group was not a source of heterogeneity. Regardless of whether the sample size per group was ≥50, the edaravone dexborneol group demonstrated significant benefit, with statistical significance.

Subgroup analysis revealed that differences in baseline NIHSS scores and onset time within 24 h may be sources of heterogeneity, whereas differences in control group treatment modalities, reperfusion therapy, randomized enrollment, and a sample size of ≥50 per group were not likely sources of heterogeneity.

Given the reliability of data from randomized enrollment and larger sample sizes, a combined analysis of randomized enrollment plus a sample size of ≥50 per group was performed (Figure 4G). This analysis included 18 studies: 5,639 patients in the edaravone dexborneol group vs. 3,998 in the control group. [*I*^2^ = 96%, MD = –2.03, 95% CI:–2.80 to–1.26, *p* < 0.00001]. Despite high heterogeneity, this result further confirmed a statistically significant benefit of edaravone dexborneol in the treatment of cerebral infarction.

Given the urgency of reperfusion therapy for ultra-early cerebral infarction and its marked improvement in outcomes, a meta-analysis was conducted in studies with a sample size of ≥50 per group, randomized enrollment, and reperfusion therapy (Figure 4H). This analysis included 5 studies: 933 patients in the edaravone dexborneol group vs. 915 in the control group [*I*^2^ = 95%, MD = –2.29, 95% CI:–3.96 to–0.63, *p* < 0.00001]. Despite high heterogeneity, the results demonstrated a statistically significant benefit of edaravone dexborneol in treating ultra-early cerebral infarction. A meta-analysis was also performed in studies with a sample size of ≥100 per group (Figure 4I). This analysis included 5 studies: 4,848 patients in the edaravone dexborneol group vs. 3,206 in the control group [*I*^2^ = 87%, MD = –0.72, 95% CI:–1.34 to–0.14, *p* < 0.00001]. Despite high heterogeneity, these findings further confirmed a statistically significant benefit of edaravone dexborneol in the treatment of cerebral infarction.

#### 3.3.3 Efficacy of edaravone dexborneol-changes in mRS before and after treatment

**Figure 5:**
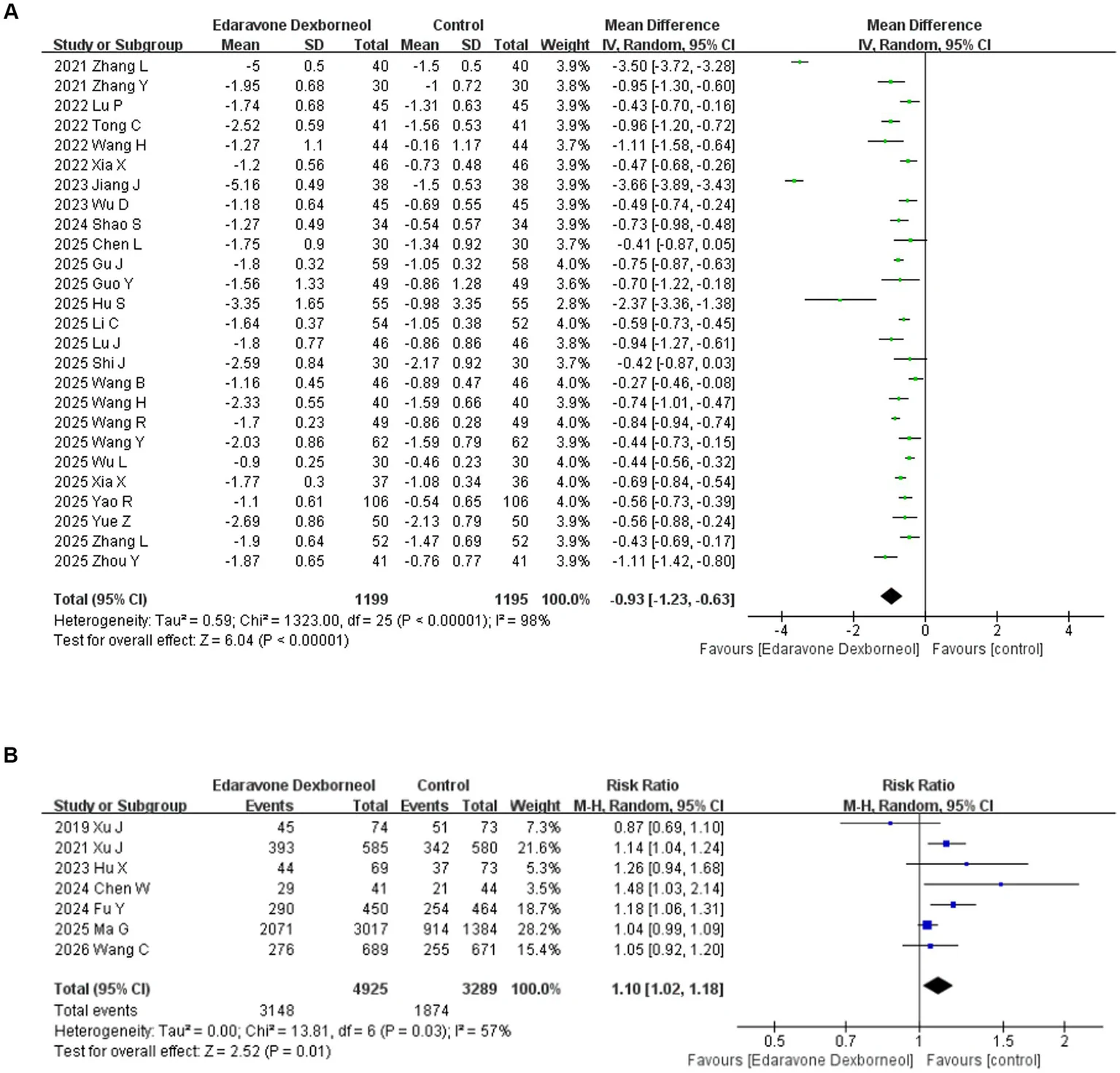
**(A)** Forest plot of pre- and post-treatment mRS changes at 2 weeks. **(B)** Forest plot of the proportion of patients with mRS 0–1 at 3 months after treatment.

Figure 5A presents the efficacy evaluation of mRS changes before and after 2 weeks of treatment. This analysis included 26 studies: 1,199 patients in the edaravone dexborneol treatment group vs. 1,195 in the control group. [I^2^ = 98%, MD = −0.93, 95% CI: −1.23 to −0.63, p < 0.00001]. Despite the high heterogeneity, the results further confirmed that edaravone dexborneol significantly improved mRS scores at 2 weeks after treatment for cerebral infarction, with a statistically significant difference. Figure 5B shows the proportion of patients achieving an mRS score of 0–1 at 90 days after treatment. This analysis included 7 studies, 4,925 patients in the edaravone dexborneol treatment group vs. 3,289 in the control group, [*I*^2^ = 57%, RR = −1.10, 95% CI: 1.02 to 1.18, *p* = 0.01]. The results indicated that Edaravone Dexborneol treatment for acute cerebral infarction led to a statistically significant improvement in functional outcome at 3 months.

### 3.4 Safety analysis

**Figure 6:**
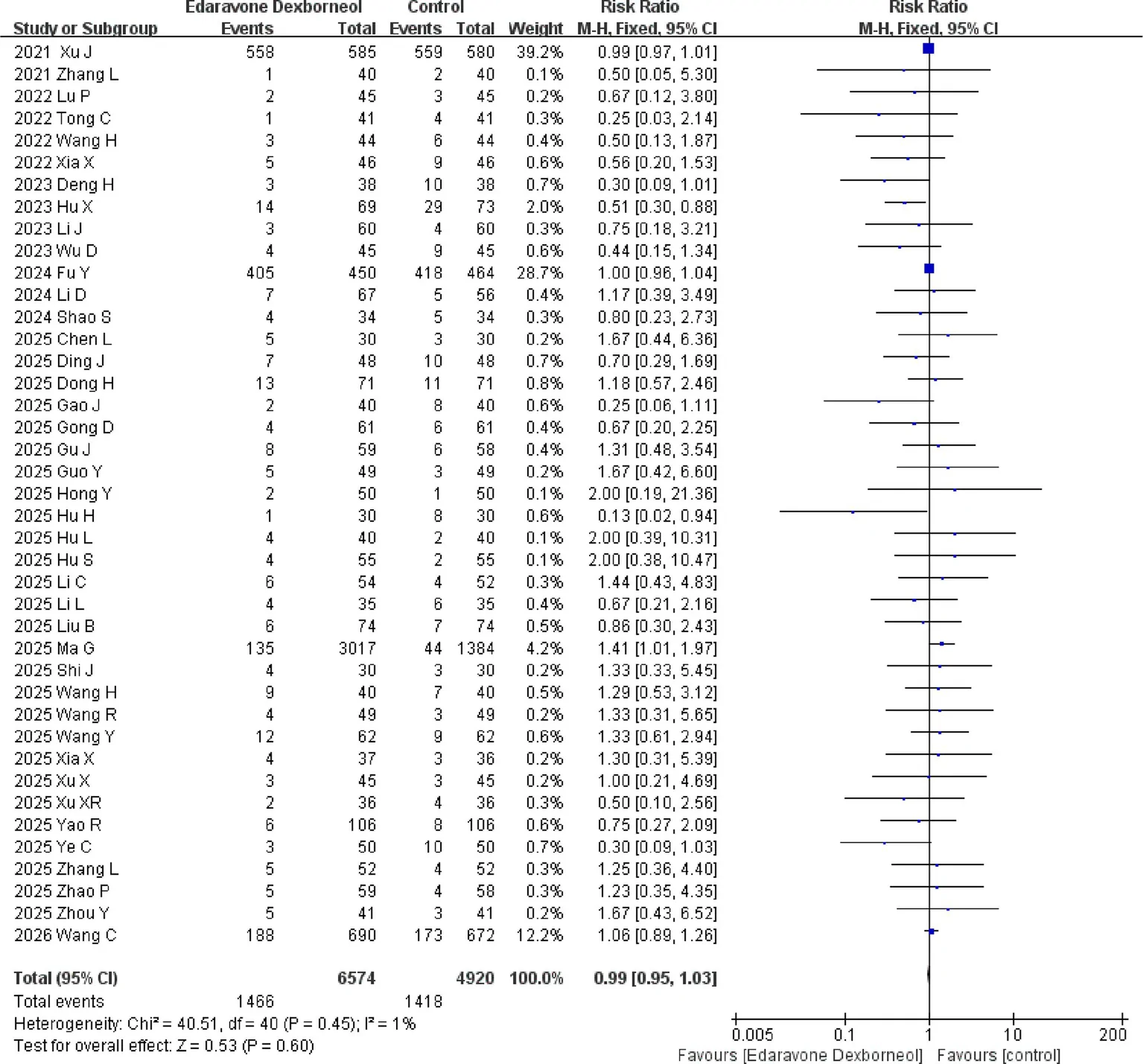
Forest plot of adverse event incidence after treatment for safety evaluation.

Forty-one of the 54 included studies reported the incidence of adverse reactions during hospitalization or within 3 months. Due to low heterogeneity (*I*^2^ = 1%), a fixed-effects model was applied. The results indicated that the incidence of adverse reactions was slightly lower in the edaravone dexborneol group (22.30%) compared to the control group (28.82%). There was no statistically significant difference in the incidence of adverse reactions between the two groups [RR = 0.99, 95% CI = 0.95 to 1.03, *p* = 0.60] (Figure 6).

Among the 54 included studies, 13 studies (involving approximately 10% of patients) did not report adverse events or only stated ‘no adverse events occurred’ without providing the exact number of cases, and thus could not be included in the pooled safety analysis. The remaining 41 studies provided complete adverse event data and were included in the safety analysis.

### 3.5 Publication bias and sensitivity analysis

**Figure 7:**
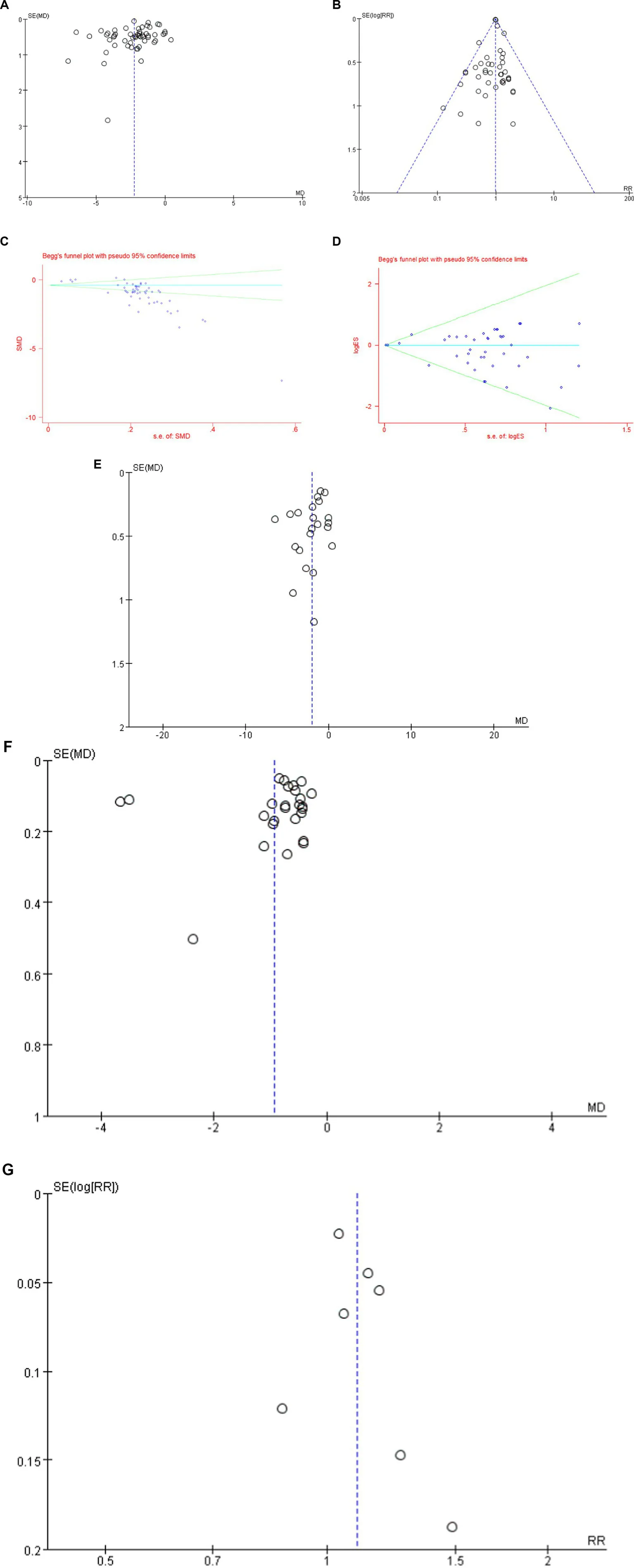
**(A)** Funnel plot for studies reporting change in NIHSS score. **(B)** Funnel plot for safety analysis. **(C)** Begg’s funnel plot for studies reporting change in NIHSS score. **(D)** Begg’s funnel plot for safety analysis. **(E)** Funnel plot of data from studies with a sample size of ≥50 per group. **(F)** Funnel plot of mRS changes at 2 weeks after treatment. **(G)** Funnel plot of the proportion of patients with mRS 0–1 at 3 months after treatment.

The funnel plot for the 54 studies reporting NIHSS change (Figure 7A) and Begg^,^s test (Figure 7C) (*p* = 0.000) indicated the presence of publication bias. In contrast, the funnel plot for safety analysis (Figure 7B) and Begger’s test (Figure 7D) (*p* = 0.195) showed no significant publication bias. The publication bias for NIHSS changes was high. After excluding studies with a sample size of less than 50, a publication bias test was performed, and the funnel plot was largely symmetric, suggesting low publication bias (Figure 7E). The funnel plot for the change in mRS before and after 2 weeks of treatment indicated low publication bias (Figure 7F). Similarly, the funnel plot for the proportion of patients with mRS 0–1 at 3 months after treatment also showed low publication bias (Figure 7G).

**Figure 8:**
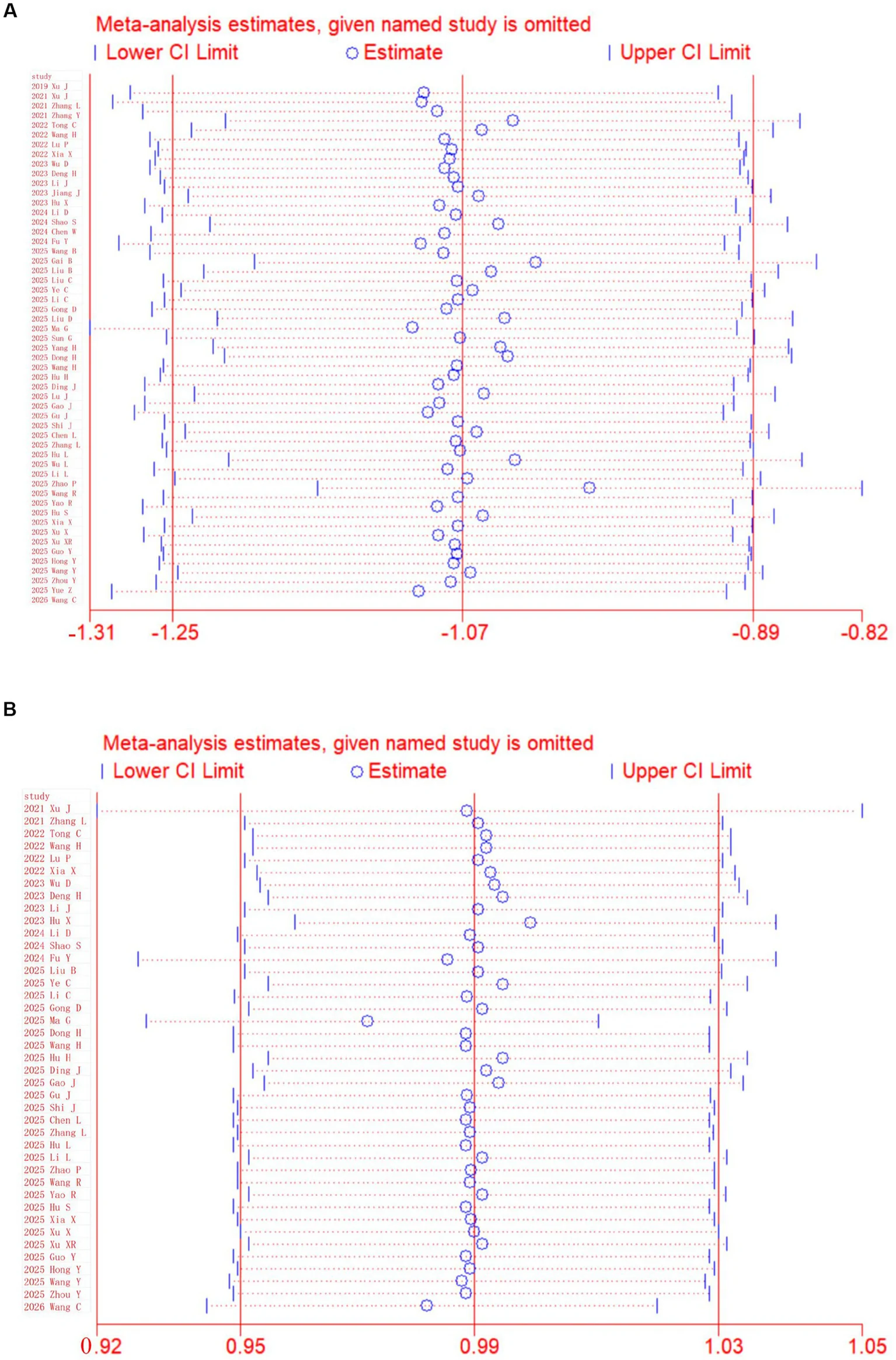
**(A)** Sensitivity analysis for the change in NIHSS score with edaravone dexborneol treatment. **(B)** Sensitivity analysis for safety outcomes.

Sensitivity analysis performed by sequentially removing each study (Figures 8A,B) revealed no substantial change in the overall results, indicating the stability of the findings.

### 3.6 GRADE assessment

The present review employed the Grading of Recommendations, Assessment, Development, and Evaluations (GRADE) framework to assess the certainty of evidence for the evaluated outcomes. The certainty of evidence varied, ranging from high to very low. For the primary outcome-change in the National Institutes of Health Stroke Scale (NIHSS) score—the evidence was rated as low due to the included randomized controlled trials and cohort studies having an overall low quality with respect to risk of bias. For the change in mRS as an efficacy endpoint and the incidence of adverse reactions for safety assessment, the evidence was rated as moderate due to the included randomized controlled trials and cohort studies having an overall moderate quality with respect to risk of bias. Overall, the strength of the evidence was downgraded based on factors including risk of bias, inconsistency, indirectness, imprecision, and publication bias, highlighting the need for further high-quality studies to confirm these findings.

## 4 Discussion

Acute ischemic stroke (AIS) is a leading cause of global morbidity and mortality (70). Timely and effective treatment is crucial for patient prognosis. Cerebral ischemia triggers a complex cascade involving multiple pathways such as oxidative stress, calcium overload, inflammation, blood–brain barrier disruption, excitotoxicity, and mitochondrial dysfunction (71). Cerebroprotective agents aim to intervene at different stages of this ischemic cascade, thereby mitigating neuronal damage and salvaging the ischemic penumbra. Given the complexity of ischemic injury, where multiple damaging pathways interact and form a vicious cycle, single-target agents often fail to achieve the desired therapeutic effect (72). In 2019, the Academic Industry Roundtable from Stroke Treatment Advancement introduced a new multi-target protection concept, emphasizing the importance of simultaneously targeting multiple pathways for neuroprotection (5). Consequently, multi-target drugs that exert pleiotropic effects by intervening at several points in the ischemic cascade have become a key strategy in AIS treatment (72). Edaravone has demonstrated efficacy in reducing neuronal and endothelial damage through free radical scavenging (12, 73, 74). Edaravone dexborneol, formulated in a 4:1 ratio of edaravone to borneol, can additionally inhibit the proinflammatory activation of microglia/macrophages and astrocytes, producing a synergistic therapeutic effect (75). These mechanisms plausibly explain the beneficial effects of edaravone dexborneol observed in this large-scale meta-analysis. The agent is available in intravenous and sublingual formulations, with pharmacokinetic data indicating similar total bioavailability for both (11). Although edaravone dexborneol is widely used for AIS in China, comparative studies on its efficacy and safety remain limited.

Haobo Gao et al. (14) conducted a meta-analysis including five English-language articles up to December 2024. Five of those articles reported NIHSS scores, with substantial heterogeneity among studies (*I*^2^ = 99%, *p* < 0.001); therefore, a random-effects model was used. The results showed that the NIHSS score was lower in the experimental group than in the control group (95% CI: −4.67 to −0.13, *p* = 0.04). Four articles reported safety outcomes, with no heterogeneity (*I*^2^ = 0%, *p* = 0.63), and a fixed-effect model was adopted. The analysis revealed no statistically significant difference in the incidence of adverse reactions between the experimental and control groups (95% CI: 0.67 to 1.04, *p* = 0.11). It was concluded that edaravone dexborneol is safe and offers short-term improvements in neurological function in patients with acute ischemic stroke (AIS), although its long-term clinical value remains uncertain. In contrast, Khaled Moghib et al. (15) included seven studies up to December 30, 2024. The pooled analysis of functional outcomes at 90 days, based on five studies, demonstrated a significant benefit, with a 39.5% higher likelihood of achieving favorable mRS scores (OR = 1.40, 95% CI: 1.18–1.65, *p* = 0.0001), without evidence of heterogeneity (*I*^2^ = 0%). In contrast, the pooled analysis of NIHSS outcomes across seven studies using a random-effects model was not significant (SMD = −0.113, 95% CI: −0.333 to 0.107, *p* = 0.314), with substantial heterogeneity (*I*^2^ = 72.7%). This meta-analysis concluded that edaravone dexborneol exhibits considerable potential as a neuroprotective agent in the context of acute ischemic stroke, providing both functional and cognitive advantages, alongside a favorable safety profile. However, the results indicate that edaravone dexborneol did not significantly improve short-term NIHSS scores in acute ischemic stroke, but instead showed a significant improvement in neurological function after 90 days. Kashbour et al. (76) included five studies up to October 12, 2024. The results showed that ED significantly improved the odds of achieving mRS ≤ 1 at 90 days (OR = 1.47, 95% CI [1.25–1.74], *p* < 0.00001). While changes in NIHSS score at 14 days were not significant (*p* = 0.35), ED showed significant improvement at 30 days (MD = −1.77, 95% CI [−2.82 to −0.72], *p* = 0.0009). ED also significantly reduced the risk of hemorrhagic transformation (OR = 0.47, 95% CI [0.24–0.92], *p* = 0.03). The conclusion was that ED demonstrates significant neuroprotective benefits in AIS by improving functional outcomes and reducing the risk of hemorrhagic transformation. This meta-analysis also indicated that ED improves neurological function at 30 and 90 days after cerebral infarction, but did not show significant short-term functional outcomes. Zhong W et al. (77) included 19 studies (in both Chinese and English) up to December 31, 2024. The results showed that after 2 weeks of treatment for acute ischemic stroke, compared with edaravone as the control, edaravone dexborneol demonstrated superior efficacy in reducing NIHSS scores (*I*^2^ = 92%, MD = −1.92, 95% CI: −2.61 to −1.23, *p* < 0.0001) and mRS scores (*I*^2^ = 99%, MD = −1.69, 95% CI: −2.49 to −0.90, *p* < 0.0001). Safety analysis indicated that the edaravone dexborneol group had a lower incidence of adverse reactions during treatment (*I*^2^ = 0%, RR = 0.47, 95% CI: 0.33 to 0.69, *p* < 0.0001). The authors concluded that edaravone dexborneol was superior to edaravone alone in both efficacy and safety. This study suggests that edaravone dexborneol provides short-term benefits for treating cerebral infarction but lacks evaluation of long-term outcomes.

This meta-analysis included 54 studies. Regarding efficacy evaluation, the edaravone dexborneol treatment group showed a reduction of 2.27 in NIHSS score compared with the control group, with a statistically significant difference (*I*^2^ = 93%, MD = −2.27, 95% CI: −2.61 to −1.94, *p* < 0.00001) (Figure 3). The NIHSS score can serve as a predictor of both short- and long-term health utility values in patients with AIS; each 1-point increase in NIHSS score was associated with a decrease in utility value of 0.029 at 1 month and 0.031 at 5 years (78). In the subgroup analysis, based on the mean baseline NIHSS score, patients were categorized into mild-to-moderate, moderate-to-severe, and severe stroke subgroups for analysis. For the mild-to-moderate stroke subgroup: *I*^2^ = 91%, MD = −1.92, 95% CI: −2.30 to −1.54, *p* < 0.00001. For the moderate-to-severe stroke subgroup: *I*^2^ = 94%, MD = −2.76, 95% CI: −3.72 to −1.79, *p* < 0.00001. For the severe stroke subgroup: *I*^2^ = 80%, MD = −3.50, 95% CI: −4.76 to −2.24, *p* = 0.04 (Figure 4A). These results suggest that the higher the baseline NIHSS score, the more pronounced the neurological recovery following edaravone dexborneol treatment (changes in NIHSS before and after treatment were −1.92, −2.76, and −3.5 for mild-to-moderate, moderate-to-severe, and severe stroke). But patients with higher baseline NIHSS scores showed larger absolute NIHSS reductions. However, this observation should be interpreted cautiously, because greater absolute improvement may partly reflect greater potential for change (regression to the mean) rather than a differential treatment effect. Therefore, we cannot conclude that more severe patients derive proportionally greater benefit.

Given the substantial heterogeneity, subgroup analyses were performed based on differences in baseline NIHSS scores before treatment (Figure 4A), whether onset time was within 24 h (Figure 4C), whether reperfusion therapy was administered (Figure 4D), differences in control group treatment regimens (Figure 4E), and whether the sample size per group was ≥50 (Figure 4F). These analyses identified differences in baseline NIHSS scores and onset time within 24 h as sources of heterogeneity. All subgroup analyses confirmed that the edaravone dexborneol treatment group had a significantly greater reduction in NIHSS score compared with the control group, with statistically significant differences. Considering the reliability of results from randomized enrollment and studies with large sample sizes, a meta-analysis combining randomized enrollment with a per-group sample size ≥50 was performed (Figure 4G). This analysis included 18 studies involving 5,639 patients in the edaravone dexborneol group and 3,998 in the control group (*I*^2^ = 96%, MD = −2.03, 95% CI: −2.80 to −1.26, *p* < 0.00001). Despite the high heterogeneity, this result similarly demonstrated a significant effect of edaravone dexborneol in treating cerebral infarction. Given the unique characteristics of the reperfusion therapy population—namely, the urgent need for early reperfusion in ultra-early cerebral infarction, where time is brain and early treatment tends to yield more favorable outcomes—a meta-analysis was performed including studies with a sample size >50, randomized enrollment, and reperfusion therapy (Figure 4H). This analysis included 5 studies with 933 patients in the edaravone dexborneol group and 915 in the control group (*I*^2^ = 95%, MD = −2.29, 95% CI: −3.96 to −0.63, *p* < 0.00001). Again, despite high heterogeneity, the result confirmed a significant effect of edaravone dexborneol in treating ultra-early cerebral infarction. Furthermore, a meta-analysis of studies with a per-group sample size ≥100 was conducted (Figure 4I), including 5 studies with 4,848 patients in the edaravone dexborneol group and 3,206 in the control group (*I*^2^ = 87%, MD = −0.72, 95% CI: −1.34 to −0.14, *p* < 0.00001). Despite the high heterogeneity, the result indicated a significant effect of edaravone dexborneol in treating cerebral infarction. Therefore, regardless of whether univariate subgroup analyses, multivariate cross-analyses, or simple large-sample meta-analyses were performed, all consistently demonstrated that edaravone dexborneol significantly reduces NIHSS scores at 2 weeks after acute ischemic stroke.

At 2 weeks after treatment, the edaravone dexborneol group showed a reduction of 0.93 in mRS score compared with the control group, with a statistically significant difference (Figure 5A) [*I*^2^ = 98%, MD = −0.93, 95% CI: −1.23 to −0.63, *p* < 0.00001]. Given the similarity between the heterogeneity analysis of the change in mRS before and after treatment and the subgroup analysis of sources of heterogeneity for the change in NIHSS before and after treatment, the sources of heterogeneity for the change in mRS at 2 weeks were considered likely related to differences in baseline NIHSS scores and onset time. Therefore, no additional subgroup analyses were performed. Regarding the proportion of patients achieving mRS 0–1 at 3 months after treatment (Figure 5B), seven studies were included, comprising 4,925 patients in the edaravone dexborneol group and 3,289 in the control group [*I*^2^ = 57%, RR = 1.10, 95% CI: 1.02 to 1.18, *p* = 0.01]. These results indicate that edaravone dexborneol significantly improved functional outcomes at 3 months after acute cerebral infarction. Thus, in evaluating the prognosis of acute ischemic stroke using mRS at 2 weeks or 90 days, edaravone dexborneol can achieve improvements in both short-term and long-term neurological function.

In terms of safety assessment, there was no statistically significant difference between the edaravone dexborneol treatment group and the control group (RR = 0.99, 95% CI: 0.95 to 1.03, *p* = 0.60).

The funnel plot for the 54 studies reporting change in NIHSS score (Figures 7A,C) indicated the presence of publication bias. In contrast, the funnel plot for the safety analysis (Figures 7B,D) showed no significant publication bias. The publication bias for the NIHSS change was relatively high; after excluding studies with a sample size smaller than 50, the funnel plot for the publication bias test appeared largely symmetrical, suggesting low publication bias (Figure 7E). The funnel plot for the change in mRS score at 2 weeks after treatment suggested low publication bias (Figure 7F), while the funnel plot for the proportion of patients achieving mRS 0–1 at 3 months showed no significant publication bias (Figure 7G).

Sensitivity analysis performed by sequentially removing each study (Figures 8A,B) revealed no substantial change in the overall results, indicating the stability of the findings. Compared with previous reviews and meta-analyses published before 2026, the present meta-analysis confirms that edaravone dexborneol has definite short- and long-term efficacy and a favorable safety profile in the treatment of acute ischemic stroke.

A recent 2026 meta-analysis also confirmed the efficacy of edaravone dexborneol in treating cerebral infarction. Sabet H et al. (79) included 13 studies, demonstrating that ED significantly improved 90-day mRS scores (0–1) compared with edaravone alone (RD: 0.08, 95% CI: [0.03, 0.13], *p* = 0.001). When compared with standard treatment, NIHSS scores were significantly lower in the EDB group (MD: −2.18, 95% CI: [−3.75, −0.62], *p* = 0.006). The authors concluded that EDB might be an effective treatment for improving functional recovery in patients with AIS and appears to have a relatively favorable safety profile. Eman Tariq et al. (80) included eight studies in a meta-analysis that assessed the change in NIHSS score from baseline to 14 days. The pooled analysis showed a modest but statistically significant improvement in the ED group compared with control (MD = −0.73; 95% CI: −1.35 to −0.11; *p* = 0.02), with substantial heterogeneity (*I*^2^ = 90%). Six studies reported the proportion of patients achieving mRS 0–1 at 90 days. Overall, ED significantly increased the likelihood of an excellent functional outcome compared with control (RR = 1.14; 95% CI: 1.07–1.20; *p* < 0.00001; *I*^2^ = 0%). Another meta-analysis of six studies found that ED did not significantly reduce the risk of death compared with control (RR = 0.91; 95% CI: 0.74–1.12; *p* = 0.38; *I*^2^ = 1%). In terms of safety, the meta-analysis revealed that ED did not significantly affect the risk of any adverse event (RR = 1.00; 95% CI: 0.97–1.02; *p* = 0.78; *I*^2^ = 0%).

This study has several limitations: (1) Some of the included studies did not describe their randomization methods, and several had small sample sizes, which may have introduced bias. (2) Adverse reactions were only assessed during the treatment period or within 3 months, and there was a lack of uniform definitions for adverse events across studies, potentially affecting the reliability of the safety results. (3) Since edaravone dexborneol was developed and is primarily used in China, all included studies involved Chinese patients, and there is a lack of clinical application data from Africa and Europe.

## 5 Conclusion

This meta-analysis provides evidence supporting the efficacy and safety of edaravone dexborneol for both short-term and long-term treatment of acute ischemic stroke. It significantly reduces NIHSS and mRS scores at 10–14 days after the acute phase, and increases the proportion of patients achieving an mRS score of 0–1 at 90 days post-treatment. Patients with higher baseline NIHSS scores may derive greater prognostic benefit from edaravone dexborneol therapy. However, large-scale, multicenter, multinational clinical studies are still needed to further validate its efficacy and safety.

## Data Availability

The original contributions presented in the study are included in the article/supplementary material, further inquiries can be directed to the corresponding author/s.
